# Omentopexy after laparoscopic sleeve gastrectomy in children and adolescents: is it effective in reducing post-operative complications?

**DOI:** 10.1007/s13304-026-02535-8

**Published:** 2026-02-13

**Authors:** Mohamed Mahfouz, Mohammad Daboos, Mohamed Abdelmaboud, Ibrahim Gamaan, Abd-Elfattah Kalmoush, Hatem Alsherbiny, Tharwat Hussien, Ahmed Azab, Mahmoud Mousa, Mohamed Emara

**Affiliations:** 1 Pediatric Surgery Department, Faculty of Medicine, Al-Azhar University, Cairo, Egypt; 2 General Surgery Department, Faculty of Medicine, Al-Azhar University, Cairo, Egypt; 3 Pediatric Surgery Unit, Department of Surgery, Al-Azhar University, Assuit, Egypt

**Keywords:** Laparoscopic sleeve gastrectomy, Pediatric obesity, Omentopexy, Gastrointestinal symptoms

## Abstract

Laparoscopic sleeve gastrectomy (LSG) is one of the most commonly performed restrictive bariatric procedures. Postoperative gastrointestinal complications are distressing in patients who have undergone this procedure. Most of these complications can be prevented by omentopexy as a modification on laparoscopic sleeve gastrectomy. The purpose of this study was to evaluate the effectiveness of omentopexy during laparoscopic sleeve gastrectomy in reducing postoperative complications. This prospective comparative study, conducted between January 2015 and October 2024 at Al-Azhar University Hospitals, encompassed 48 pediatric patients diagnosed with obesity. The patients were randomly allocated into two groups: group 1 comprised 24 patients treated with LSG with omentopexy and group 2 comprised 24 patients treated with LSG without omentopexy. Preoperative data and postoperative outcomes were collected for all patients in each group. 48 morbidly obese patients, divided into two groups of 24 each, were included in this study. Both groups were comparable in baseline characteristics including age, sex distribution, BMI, preoperative GERD-Q scores, and comorbidities. Operative times were significantly longer in the omentopexy group (64.23 ± 8.12 min) compared to the non-omentopexy group (54.15 ± 5.02 min, *P* = 0.044). The omentopexy group had significantly lower rates of nausea (8.3% vs. 20.8%), vomiting (8.3% vs. 33.3%), and regurgitation (8.3% vs. 16.6%). One case of staple line leak occurred in the non-omentopexy group, a difference that was not statistically significant. Hospital stay was significantly shorter in the omentopexy group (1.78 ± 0.858 days) compared to the non-omentopexy group (4.47 ± 1.25 days, *P* = 0.011). Both groups achieved comparable weight loss (BMI: 27.35 ± 4.57 vs. 28.85 ± 5.68) and postoperative GERD-Q scores (6.73 ± 1.86 vs. 6.85 ± 2.01), indicating similar efficacy in the treatment of obesity. Omentopexy following laparoscopic sleeve gastrectomy is associated with a significant reduction in early post-operative nausea and vomiting and a shorter hospital stay. While there were no staple line leaks in the omentopexy group, this study was not powered to definitively conclude a reduction in this complication. Also, the extent to which this technique alleviates gastroesophageal reflux disease remains uncertain.

## Introduction

The prevalence of pediatric obesity has increased significantly in recent years largely, due to behavioral and environmental factors, particularly limitations in daily activities [1, 2]. While the primary focus should be on obesity prevention, numerous treatment options exist to mitigate associated comorbidities. These include behavioral modifications, pharmacotherapy, metabolic and bariatric surgeries. However, it is important to note that lifestyle modifications and medical treatments often prove ineffective in achieving clinically significant or sustainable weight loss. Recently, Laparoscopic Sleeve Gastrectomy (LSG) has emerged as a promising intervention for the management of pediatric obesity, with initial findings suggesting its effectiveness and safety [3, 4].

In the early postoperative phase, when most LSG problems occur, gastrointestinal issues such as leakage, food intolerance, regurgitation, nausea, and vomiting are frequent. One method among many that has been proposed to lessen these multiple issues is omentopexy [5]. It was also suggested that these debilitating postoperative problems, such as vomiting, regurgitation, and food intolerance, may be caused by incorrect positioning or twisting of the gastric sleeve due to the loss of stabilization of the greater curvature of the stomach, while fixation of the gastric tube by omentopexy prevent this torsion and subsequent gastric stricture [6]. In light of this, our study aimed to assess the effectiveness of omentopexy during laparoscopic sleeve gastrectomy in reducing early gastrointestinal adverse symptoms. Furthermore, we sought to investigate potential mechanisms, including its impact on post-operative bleeding.

## Patients and methods

This prospective comparative study was conducted in Pediatric and General surgery departments in Al-Azhar University Hospitals between January 2015 and October 2024. Patients included in this study were selected according to the following inclusion and exclusion criteria: age less than 18 years, body mass index (BMI) of 40 kg/m^2^, or BMI of 35 kg/m^2^ with at least one comorbid condition, such as hypertension, diabetes, obstructive sleep apnea, and dyslipidemia. Patients with secondary obesity (as a result of hypothyroidism or cortisol therapy), syndromic obesity, monogenetic obesity, and previous bariatric operations were excluded from this study. Patients were randomly assigned to one of two groups using the sealed, closed envelope method: group 1 included patients managed with LSG with omentopexy, and group 2 included patients managed with LSG without omentopexy.

The research conducted adhered to the Helsinki Declaration’s principles and received approval from the Ethics Committee at Al-Azhar University’s Faculty of Medicine *(registration number*: *2/2015OBSGN63)* and registered at https://clinicaltrials.gov/ with ID *(NCT06811623)*. Informed consent forms were signed by patients’ caregivers prior to surgical procedures. A thorough medical history and physical examination, with obesity assessment utilizing the WHO growth chart for BMI were performed. Blood tests were also conducted to measure lipid profiles, liver enzymes, thyroid hormones, cortisol levels, complete blood count, coagulation profiles, and HbA1C. Additional diagnostic procedures, such as respiratory function tests and abdominal ultrasounds, were also carried out.

**Sample size calculation 48**: Population size 50, population proportion 54%, Confidence level 95%, Margin of Error 5%.

### Method of randomization and patient selection

The sample size was determined prior to the commencement of the study. Subsequently, 48 opaque envelopes were prepared, each containing a card. Of these 48 envelopes, 24 contained cards designated for “Sleeve gastrectomy with omentopexy” and the remaining 24 envelopes contained cards designated for “Sleeve gastrectomy without omentopexy.” All envelopes were sealed and randomized. Patients were individually presented, and each patient’s parent selected one of the sealed envelopes without prior knowledge of its contents. The surgical committee unsealed the envelopes to ascertain the selected technique. This methodology is referred to as the permuted block randomization design, which is employed to ensure equal and balanced distribution of participants between both groups in the present study.

## Surgical procedure

**Fig. 1:**
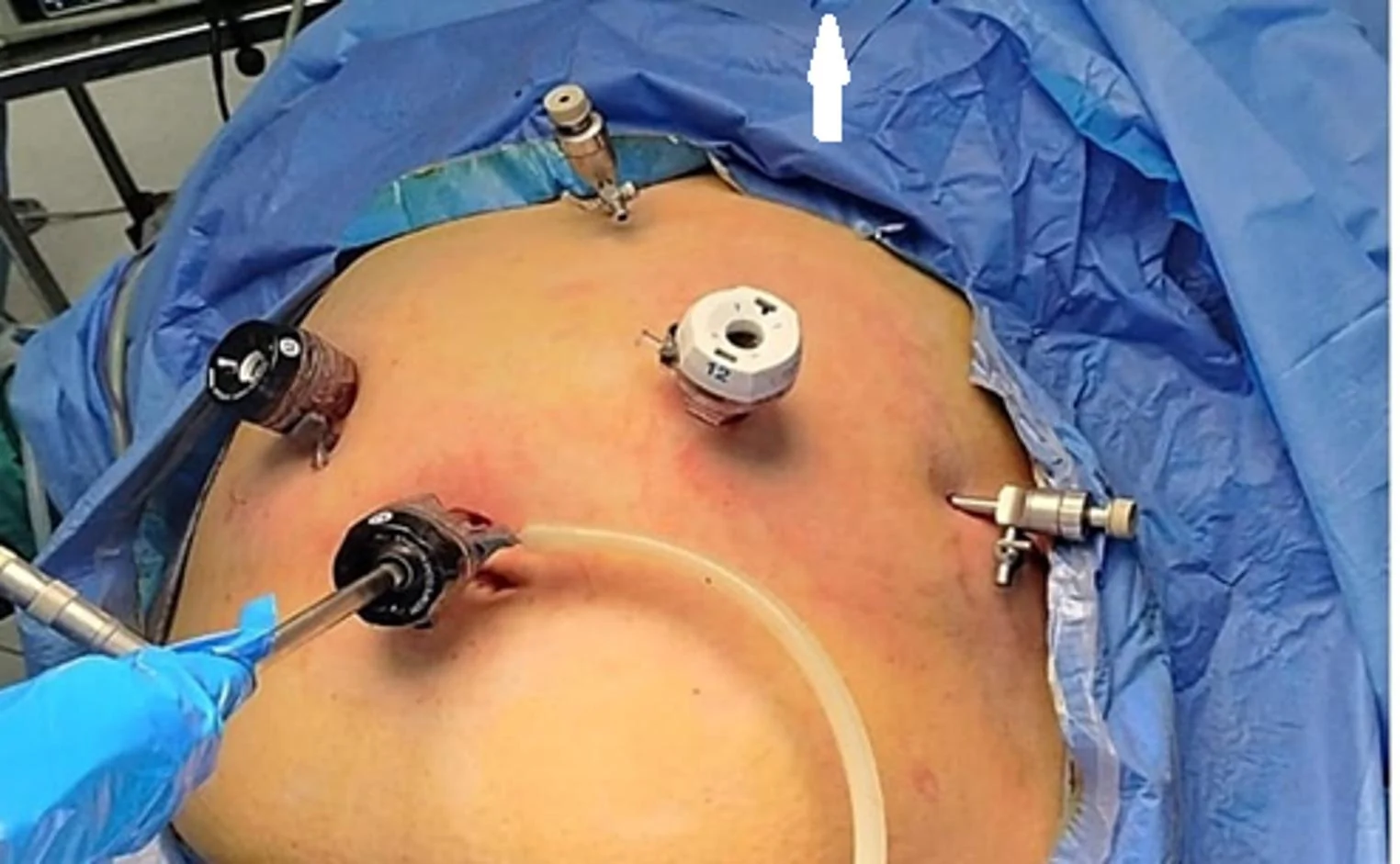
Ports sites (white arrow point to the head of the patient)

**Fig. 2:**
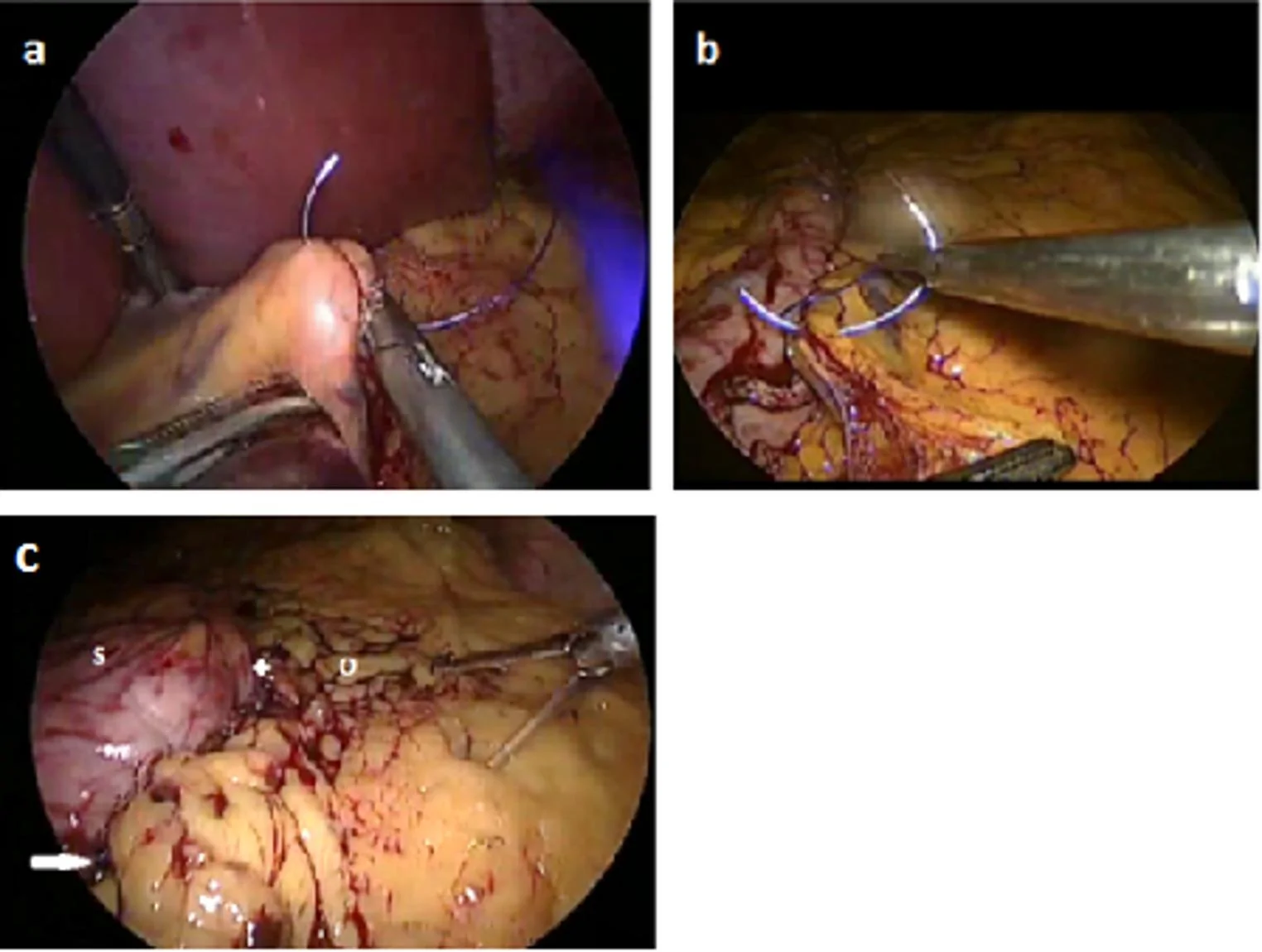
**a** 1st stitch in omentopexy. **b** during omentopexy. **c** Omentopexy was completed. White arrow, the site of ending of omentopexy. S, stomach. O, omentum. White star is the suture line

With the patient in the supine position, under general anesthesia, and following sterilization and draping, five ports were inserted (Fig. 1). The patient was positioned in the reversed Trendelenburg position, wherein the principal steps were as follows: (a) the greater omentum was dissected, and the short gastric vessels were ligated utilizing a LigaSure device; (b) 32–36 French orogastric bougie was introduced into the pylorus; (c) stapling commenced 3 to 4 cm proximal to the pylorus and continued until the angle of His was reached. In group 1, omentopexy was performed in addition to the standard LSG, extending from 4 cm distal to the gastroesophageal junction to 4 cm proximal to the pylorus, utilizing a PDS 2/0 running suture (Fig. 2). Subsequently, the patients underwent intraoperative testing for leak via methylene blue injection into the stomach.

## Data collection

Preoperative demographic and clinical data were collected, including age, sex, BMI, and comorbidities, such as diabetes mellitus, obstructive sleep apnea, and hypertension. In addition, the preoperative symptoms of GERD were assessed and quantified using the GERD-Q [7]. This tool examines symptoms compatible with GERD (heartburn, acid and food regurgitation) by measuring their intensity and frequency. The score for each symptom was multiplied (intensity × frequency), leading to a final score between 0 and 18. Intraoperative data were collected, including intraoperative bleeding; operative time; early postoperative complications including leak, nausea, vomiting, and regurgitation; postoperative ondansetron requirement; days of hospital stay; and time of return to normal activities. Postoperative hemoglobin (Hb) levels were measured on the first postoperative day (POD1) in all patients to assess surgical blood loss. During the postoperative follow-up period, which ranged from 12 to 18 months, BMI and GERD-Q scores were evaluated for all patients.

### Statistical analysis

Data were collected and entered into a computer using the SPSS (Statistical Package for Social Science [SPSS] program for statistical analysis [version 21] [IBM Corp., released 2012]). The results were considered statistically significant when the probability of error was less than 5% (*P* ≤ 0.05).

## Results

48 morbidly obese patients were enrolled in this study between January 2015 and October 2024 at Al-Azhar University Hospitals. They were allocated into two groups regarding LSG with or without omentopexy, 24 patients in each group. The mean age in group 1 was 13.41 ± 0.837 years, while in group 2 the mean age was 13.52 ± 0.769 years. Group 1 included 6 (25%) male and 18 (75%) female participants, while group 2 included 4 (16.6%) male and 20 (83.4%) female participants. The mean BMI was 42.12 ± 5.34 in group 1 and 44.25 ± 6.12 in group 2, and preoperative GERD-Q scores were 12.68 ± 1.43 and 12.73 ± 1.25 in groups 1 and 2, respectively. The associated comorbidities in group 1 were reported as 4 cases (16.6%) of DM, 4 cases (16.6%) of obstructive sleep apnea, and 1 case (8.3%) of hypertension. Group 2 included 1 case (8.3%) of DM, 3 cases (21.4%) of obstructive sleep apnea, and 1 case (8.3%) of hypertension. There were no significant differences between the two groups regarding age, sex, BMI, GERD-Q, and different comorbidities, (Table 1.).

**Table 1:** Comparison between 2 groups regarding demographic and clinical data

	**Group 1** **Omentopexy** **(n=24)**	**Group 2** **Non-Omentopexy** **(n=24)**	**P-value**
** Age** (years)	13.41 ± 0.837	13.52 ± 0.769	0.720
**Sex**			
· Male · Female	6 (25%) 18 (75%)	4 (16.6%) 20 (83.4%)	0.622
**Preoperative data**			
· Preoperative BMI (kg/m^2^)	42.12 ± 5.34	44.25 ± 6.12	0.336
· Preoperative GERD-Q	12.68 ± 1.43	12.73 ± 1.25	0.822
**Comorbidities**			
· DM · Obstructive sleep apnea · Hypertension	4 (16.6%) 4 (16.6%) 1 (8.3%)	1 (8.3%) 3 (21.4%) 1 (8.3%)	0.541 0.622 0.1

BMI: Body mass index, GERD: Gastroesophageal Reflux Disease, DM: Diabetes Mellitus

Patients undergoing Omentopexy experienced significantly longer operative times compared to those without the procedure. The mean operative duration was 64.23 ± 8.12 min for group 1 and 54.15 ± 5.02 min for group 2 (*P*-value = 0.044). Intraoperative bleeding was absent in group 1, while only one case in group 2 exhibited suture line bleeding. Complications in group 1 included 2 (8.3%) cases each of nausea, vomiting, and regurgitation, with no instances of leakage. Group 2 reported higher complication rates: 5 (20.8%) cases of nausea, 8 (33.3%) cases of vomiting, 4 (16.6%) cases of regurgitation, and one case of postoperative staple line leakage (4.2%); this difference in leak rate was not statistically significant compared to the omentopexy group (0%) (*p* = 0.315). The mean Hb drop (preoperative Hb minus POD1 Hb) was significantly lower in the omentopexy group (1.2 ± 0.4 g/dL) compared to the non-omentopexy group (1.8 ± 0.6 g/dL) (*p* < 0.001).

The increase in vomiting cases in group 2 was statistically significant. All cases of nausea, vomiting, and regurgitation responded well to Ondansetron treatment. Consequently, Ondansetron was administered to 17 (70.8%) cases in group 2, whereas only 6 cases in group 1 required antiemetic (ondansetron) medication, (Table 2.). The single case of leakage in group 2 was managed through laparoscopic endo clipping.

**Table 2:** Comparison between 2 groups regarding operative and early post-operative data

	**Group 1** **Omentopexy** **(n=24)**	**Group 2** **Non-Omentopexy (n=24)**	**P-value**
**Intraoperative data**			
Operative time (min)	62.23 ± 8.12	54.15 ± 5.02	0.044*
Bleeding	0	1 (8.3%)	0.311
**Post-operative data**			
Leak Nausea Vomiting Regurgitation Postop. Ondansetron	0 2 (8.3%) 2 (8.3%) 2 (8.3%) 6(25.0%)	1(8.3%) 5 (20.8%) 8 (33.3%) 4 (16.6%) 17 (70.8%)	0.311 0.221 **0.041** * 0.285 0.003*
** Mean Hb drop (g/dl)**	1.2 ± 0.4	1.8 ± 0.6	<0.001*
** Hospital stay (days)**	1.78 ± 0.858	4.47 ± 1.25	0.001*
** Time to return normal activity (days)**	11.12 ± 6.54	14.52 ± 7.12	0.201

*: Significant difference

Patients in group 1 experienced a shorter post-operative hospital stay (1.78 ± 0.858 days) compared to those in group 2 (4.47 ± 1.25 days), with a significantly longer duration observed in patients without Omentopexy versus those with Omentopexy.

During the post-operative follow-up period 12–18 months, both groups demonstrated successful and comparable weight reduction. The BMI in group 1 was 27.35 ± 4.57, while in group 2 it was 28.85 ± 5.68. The Postoperative GERD-Q scores were 6.73 ± 1.86 for group 1 and 6.85 ± 2.01 for group 2, (Table 3.).

**Table 3:** Comparison between 2 groups regarding BMI and post-operative GERD-Q data

	Group 1 Omentopexy (n=24)	Group 2 Non-Omentopexy (n=24)	P-value
** Postoperative BMI at 12**^**th**^ **month**	27.35 ± 4.57	28.85 ± 5.68	0.448
** Postoperative GERD-Q**	6.73 ± 1.86	6.85 ± 2.01	0.871

GERD-Q: Gastroesophageal reflux disease Questionnaire

## Discussion

Pediatric obesity is associated with complications affecting nearly every system of the body, including the endocrine, gastrointestinal, pulmonary, cardiovascular, and musculoskeletal systems. The severity of these complications typically correlates with the severity of obesity [2], making LSG one of the most commonly performed procedures for obesity worldwide. Recently, it has become more favorable than Roux-en-Y gastric bypass due to its comparable efficacy and lower complication rate. However, owing to restrictive changes in gastric anatomy and increased intraluminal pressure, LSG is associated with an increased risk of postoperative regurgitation, vomiting, and other gastrointestinal symptoms [8]. A meta-analysis conducted by Zarzycki et al. [9] compared LSG with omentopexy reinforcement and LSG without reinforcement and reported that postoperative morbidities such as gastric leak and length of hospital stay were significantly reduced with omentopexy modification, suggesting that LSG with omentopexy may be a feasible procedure to reduce these morbidities. They also recommended that the procedure should be further investigated in randomized controlled trials to draw solid conclusions. Thus, the current study considered the previous recommendation and provided more relevant results, confirming the effect of omentopexy on reducing postoperative gastrointestinal morbidities.

In the present study, operative time was significantly longer in patients with omentopexy; operative time by minutes was 64.23 ± 8.12 in group 1 and 54.15 ± 5.02 in group 2 (*P* = 0.044). Consistent with these findings, a previous study by Sabry and Qassem [10] reported a significant prolongation of operative time in the omentopexy group (85 vs. 55 min in controls, *p* = 0.001), another study by Fouad et al. [11] showed similar results, the mean operative time was 72.6 and 83.4 min in non-omentopexy and omentopexy groups, respectively.

The significant reduction in early postoperative nausea, vomiting, and antiemetic requirements in the omentopexy group is a key finding of this study. Although the exact mechanism remains to be fully elucidated, several hypotheses can be proposed. First, omentopexy may provide mechanical stabilization to the newly formed gastric tube, preventing subtle torsion or kinking that could disrupt emptying and provoke symptoms, even in the absence of radiologically evident obstruction [6, 12]. Second, the suggested immunomodulatory and absorptive properties of the greater omentum may dampen the local inflammatory response and provide robust lymphatic drainage, potentially reducing edema at the gastroesophageal junction and gastric wall, thereby improving early functional recovery [5].

A further compelling explanation is provided by our analysis of postoperative hemoglobin levels, which revealed a significantly smaller Hb drop in the omentopexy group. This suggests that omentopexy acts as a hemostatic bolster, effectively tamponading microbleeding from the long staple line. Reduced postoperative blood loss and the consequent lower degree of anemia likely contribute directly to diminished symptoms, such as fatigue, dizziness, and nausea, facilitating a smoother and earlier recovery. It is plausible that these mechanisms—mechanical, inflammatory, and hemostatic—act synergistically to produce the observed clinical benefits.”

In this study, the number of patients requiring postoperative ondansetron was significantly lower in the omentopexy group. Similarly, a previous study by Sabry et al. [10] found that omentopexy improved postoperative food tolerance, as suggested by the alimentation questionnaire, which showed significantly higher postoperative food tolerance, better food scores in the omentopexy group (*P* < 0.001), and lower frequency of vomiting than in the non-omentopexy group (*P* < 0.001). As a result, patients who underwent LSG with omentopexy were more compliant with the postoperative diet plan, which in turn led to better weight loss outcomes than those who underwent standard LSG.

In the current study, the non-omentopexy group had one case of suture leakage and one case of bleeding, while the omentopexy group had no such event. This is similar to the findings of Pilone et al. [13], who reported fewer cases of leakage in the omentopexy group. In addition, in a systematic review and meta-analysis published in 2024, LSG with omentopexy showed a significantly lower rate of gastric leakage (*P* = 0.0001) and staple line bleeding (*P* = 0.00001) than LSG alone [14]. It is important to note, however, that the very low incidence of these major complications means our study was not powered to detect a statistically significant difference between groups. Larger multicenter studies are required to definitively confirm the protective effect of omentopexy against staple line leaks and bleeding.

There is a paucity of robust data regarding the impact of omentopexy on reducing the incidence of gastroesophageal reflux disease (GERD). In this study, no statistically significant differences in postoperative GERD-Q scores were observed between the study groups. This finding is consistent with a previous study by Sharma et al. [15], who also reported no significant difference in postoperative GERD symptoms between omentopexy and control groups, with incidence rates of 13.24% and 15.53%, respectively (*p* = 0.4). However, there is conflicting evidence in the literature, with some studies suggesting that omentopexy is associated with a significant reduction in the postoperative GERD scores and reflux symptoms. The potential influence of omentopexy on gastric emptying, mediated by changes in the orientation of the gastric tube, remains an area requiring further investigation [16, 17]. Additional well-designed studies are warranted to elucidate the mechanisms underlying these observations and determine the clinical efficacy of omentopexy in mitigating GERD-related outcomes.

While the present study is distinguished by its focus on pediatric bariatric surgery and omentopexy modification, it is important to acknowledge the limitations of the study. First, the relatively small sample size may reduce the statistical power to detect significant associations. Second, the assessment of GERD relied on subjective measures rather than objective endoscopic findings, which may introduce bias and affect diagnostic accuracy. These limitations highlight the need for future studies to address these shortcomings by including larger, more diverse cohorts and using standardized, objective diagnostic criteria, such as endoscopy, to validate the results and increase their robustness.

## Conclusion

Omentopexy following laparoscopic sleeve gastrectomy in children and adolescents is associated with a significant reduction in early postoperative nausea and vomiting, decreased requirement for antiemetics, and shorter hospital stay. This technique may contribute to reduced staple line bleeding, which likely underpins smoother recovery. Although no staple line leaks occurred in the omentopexy group, a definitive conclusion regarding the prevention of this complication requires further investigation with larger sample sizes. In addition, the extent to which this technique alleviates gastroesophageal reflux disease remains uncertain.

## Data Availability

The datasets used during the current study are available from the corresponding author but could not be sent owing to the medicolegal aspect of the hospital policy.
