# Correction: Omentopexy after laparoscopic sleeve gastrectomy in children and adolescents: is it effective in reducing post-operative complications?

**DOI:** 10.1007/s13304-026-02618-6

**Published:** 2026-05-11

**Authors:** Mohamed Mahfouz, Mohammad Daboos, Mohamed Abdelmaboud, Ibrahim Gamaan, Abd-Elfattah Kalmoush, Hatem Alsherbiny, Tharwat Hussien, Ahmed Azab, Mahmoud Mousa, Mohamed Emara

**Affiliations:** 1 Pediatric Surgery Department, Faculty of Medicine, Al-Azhar University Hospitals, Al-Azhar University, Cairo, Egypt; 2 General Surgery Department, Faculty of Medicine, Al-Azhar University, Cairo, Egypt; 3 Pediatric Surgery Unit, Department of Surgery, Al-Azhar University, Assuit, Egypt

**Correction to: Updates in Surgery** https://doi.org/10.1007/s13304-026-02535-8

In Tables 1 and 2 of this article, the data in the values in the columns has shifted one line up. It is not uncorrected correctly to the parameters. It has been updated. The corrected and uncorrected tables are given below.

**Corrected tables:**

**Table 1:** Comparison between 2 groups regarding demographic and clinical data

	Group 1 Omentopexy (n = 24)	Group 2 Non-Omentopexy (n = 24)	*P* value
Age (years)	13.41 ± 0.837	13.52 ± 0.769	0.720
Sex			
Male	6 (25%)	4 (16.6%)	0.622
Female	18 (75%)	20 (83.4%)	
Preoperative data			
Preoperative BMI (kg/m^2^)	42.12 ± 5.34	44.25 ± 6.12	0.336
Preoperative GERD-Q	12.68 ± 1.43	12.73 ± 1.25	0.822
Comorbidities			
DM	4 (16.6%)	1 (8.3%)	0.541
Obstructive sleep apnea	4 (16.6%)	3 (21.4%)	0.622
Hypertension	1 (8.3%)	1 (8.3%)	0.1

BMI: Body mass index, GERD: Gastroesophageal Reflux Disease, DM: Diabetes Mellitus

**Table 2:** Comparison between 2 groups regarding operative and early post-operative data

	Group 1 Omentopexy (n = 24)	Group 2 Non-Omentopexy (n = 24)	*P* value
Intraoperative data			
Operative time (min)	62.23 ± 8.12	54.15 ± 5.02	0.044*
Bleeding	0	1 (8.3%)	0.311
Post-operative data			
Leak	0	1 (8.3%)	0.311
Nausea	2 (8.3%)	5 (20.8%)	0.221
Vomiting	2 (8.3%)	8 (33.3%)	0.041*
Regurgitation	2 (8.3%)	4 (16.6%)	0.285
Postop. Ondansetron	6 (25.0%)	17 (70.8%)	0.003*
Mean Hb drop (g/dl)	1.2 ± 0.4	1.8 ± 0.6	< 0.001*
Hospital stay (days)	1.78 ± 0.858	4.47 ± 1.25	0.001*
Time to return normal activity (days)	11.12 ± 6.54	14.52 ± 7.12	0.201

*Significant difference

**Uncorrected tables:**

**Figure:**
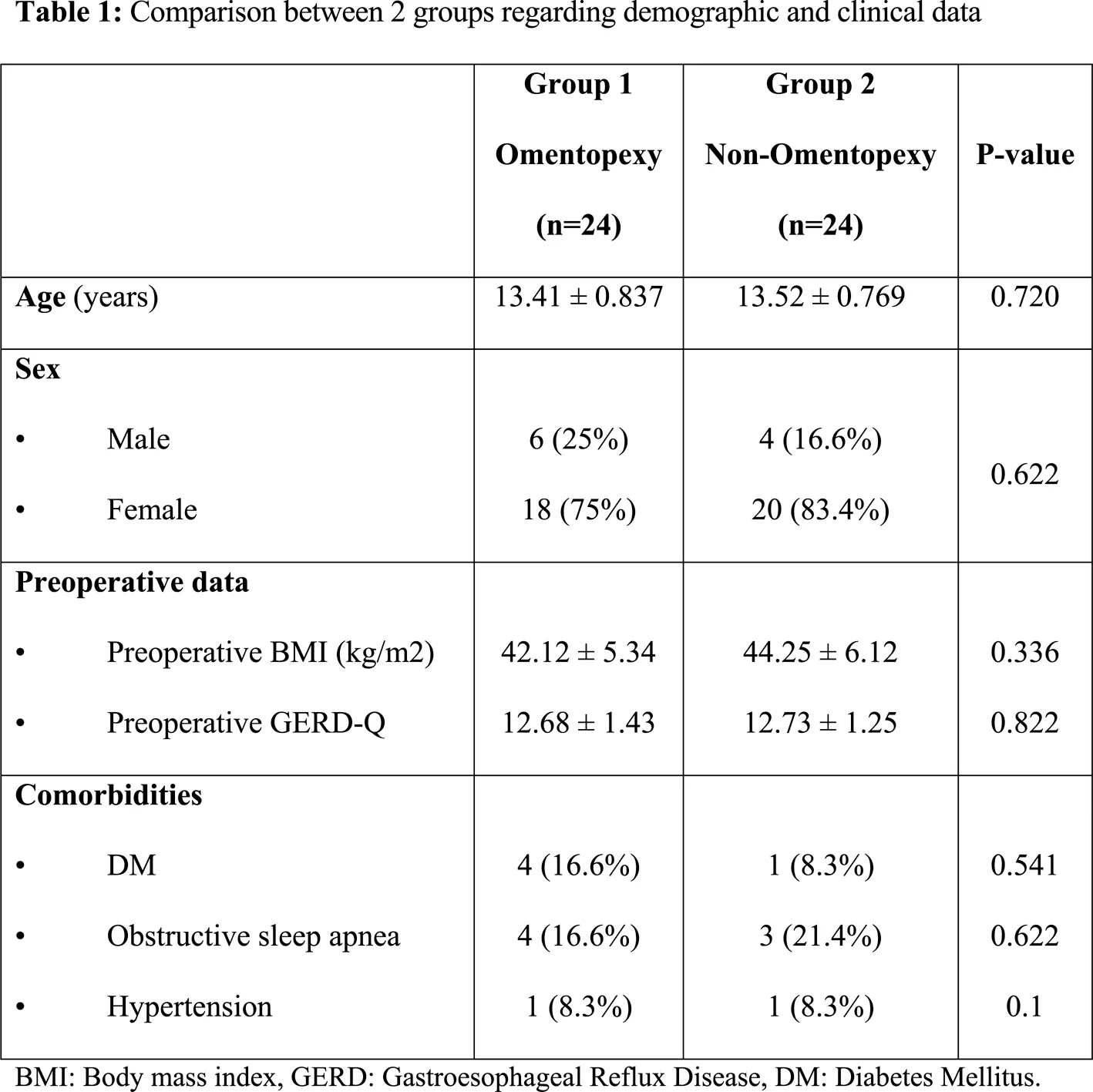

**Figure:**
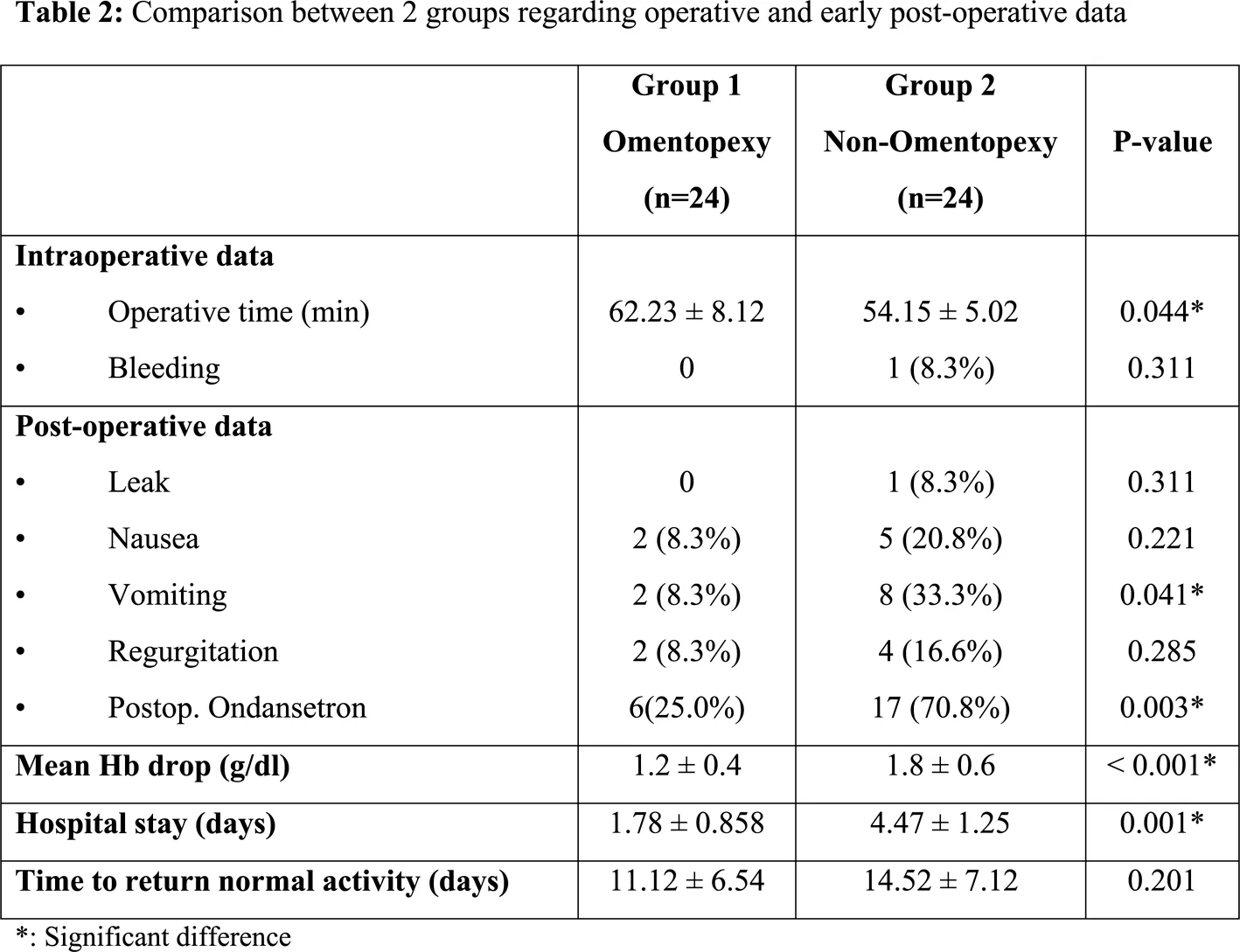

The original article has been corrected.

